# Recovery after critical illness: putting the puzzle together—a consensus of 29

**DOI:** 10.1186/s13054-017-1887-7

**Published:** 2017-12-05

**Authors:** Elie Azoulay, Jean-Louis Vincent, Derek C. Angus, Yaseen M. Arabi, Laurent Brochard, Stephen J. Brett, Giuseppe Citerio, Deborah J. Cook, Jared Randall Curtis, Claudia C. dos Santos, E. Wesley Ely, Jesse Hall, Scott D. Halpern, Nicholas Hart, Ramona O. Hopkins, Theodore J. Iwashyna, Samir Jaber, Nicola Latronico, Sangeeta Mehta, Dale M. Needham, Judith Nelson, Kathleen Puntillo, Michael Quintel, Kathy Rowan, Gordon Rubenfeld, Greet Van den Berghe, Johannes Van der Hoeven, Hannah Wunsch, Margaret Herridge

**Affiliations:** 1 Medical Intensive Care Unit, Hôpital Saint-Louis, ECSTRA team, Biostatistics and clinical epidemiology, UMR 1153 (Center of Epidemiology and Biostatistics Sorbonne Paris Cité, CRESS), INSERM, Paris Diderot Sorbonne University, Paris, France; 2 Erasme Hospital, Université libre de Bruxelles, Brussels, Belgium; 3 The University of Pittsburgh School of Medicine, Pittsburgh, PA USA; 4 King Abdullah International Medical Research Center, King Saud Bin Abdulaziz University for Health Sciences, Riyadh, Saudi Arabia; 5 St. Michael’s Hospital, Toronto, ON Canada; 6 Department of Surgery and Cancer Imperial College, London, UK; 7 Università Milano Bicocca, Milano, Italy; 8 McMaster University Medical Center, Hamilton, ON Canada; 9 Cambia Palliative Care Center of Excellence, Seattle, WA USA; 10 Toronto General Research Institute, University of Toronto, Toronto, ON Canada; 11 Vanderbilt University School of Medicine, and TN Valley Veteran’s Affairs Geriatric Research Education Clinical Center (GRECC), Nashville, TN USA; 12 The University of Chicago, Chicago, IL USA; 13 Perelman School of Medicine, Philadelphia, PA USA; 14 St Thomas Hospital, London, UK; 15 Intermountain Medical Center, Murray, UT USA; 16 Psychology Department and Neuroscience Center, Brigham Young University, Provo, UT USA; 17 University of Michigan Health System, and Ann Arbor Veterans Affairs Healthcare System, Ann Arbor, MI USA; 18 Saint-Eloi Hospital, Montpellier, France; 19 University of Brescia at Spedali Civili, Brescia, Italy; 20 Mount Sinai Hospital, Toronto, ON Canada; 21 Johns Hopkins University School of Medicine, Baltimore, MD USA; 22 Memorial Sloan Kettering Cancer Center, and Weill Cornell Medical College New York, New York, NY USA; 23 University of California, San Francisco, CA USA; 24 University Hospital Göttingen, Göttingen, Germany; 25 Intensive Care National Audit & Research Centre, London, UK; 26 Sunnybrook Health Sciences Centre, Toronto, ON Canada; 27 University Hospital Leuven, Leuven, Belgium; 28 Radboud University Nijmegen Medical Centre, Nijmegen, Netherlands; 29 Toronto General Research Institute, University of Toronto, UHN - University Health Network, Toronto, ON Canada

**Keywords:** Mechanical ventilation, Sedation, Delirium, Weakness, Intensive care, Muscular disorder, Cognitive dysfunction, Depression, Traumatic stress

## Abstract

In this review, we seek to highlight how critical illness and critical care affect longer-term outcomes, to underline the contribution of ICU delirium to cognitive dysfunction several months after ICU discharge, to give new insights into ICU acquired weakness, to emphasize the importance of value-based healthcare, and to delineate the elements of family-centered care. This consensus of 29 also provides a perspective and a research agenda about post-ICU recovery.

## Background

Over the past 25 years there has been a burgeoning critical care literature addressing the issue of longer-term outcomes, including survival, quality of life, morbidity, functional status, joblessness, and costs of care for ICU survivors [1]. Attention has also focused on ICU-acquired weakness as a generalized neuromuscular disorder that may dominate the longer-term trajectory, increase ventilatory dependence, and impede survival and ability to return to baseline functional status [2, 3]. These observations have prompted studies of mobilization at an early stage of critical illness and post-critical illness rehabilitation. The international critical care community has become increasingly aware that bed rest and immobilization in patients with sepsis or respiratory or multiple organ failure and post-ICU inflammatory states are associated with skeletal muscle wasting and increased weakness and impaired longer-term physical and neurocognitive function. In this review, we seek to highlight how critical illness and critical care (for example, sedation practices) affect longer-term outcomes, to underline the contribution of ICU delirium to cognitive dysfunction several months after ICU discharge, to give new insights into ICU acquired weakness, to emphasize the importance of value-based healthcare, and to delineate the elements of family-centered care. Longer-term outcomes are analyzed as the result of both the acute illness (pneumonia, trauma, stroke, anoxic brain injury, myocardial infarction, spinal cord injury, burn, etc.) and the critical care experience and related burden. It is likely that most of the deleterious effects of critical care also apply to acute care, with regard to both the prevalence and the mechanisms that lead to weakness, delirium, cognitive impairment, worsening of chronic organ dysfunction, or any other significant sequels. The severity of acute illness determines the degree of impairment (with age and length of ICU stay) and the chronic disease status determines the trajectory of recovery.

### Longer-term outcomes

There is no doubt that critical illness causally affects long-term outcomes. However, additional research is warranted to account for existing gaps and limitations in the literature. For example, age, frailty, and comorbidities are risk factors for critical illness but also modulate its longer-term impact. By collecting data on longer-term morbidity without being aware of pre-existing comorbidities, studies often provide a biased picture of ICU survivors. Validation of proxy reports may be important to understand pre-ICU status, such as the physical aspect of frailty, cognitive and mental health, as well as psychological and social status, and identify adequate targets for improvement [4]. Premorbid physical, cognitive, and mental health status must be considered for patients and caregivers. Studies report that, regardless of age, appreciable numbers of survivors of critical illness have acute severe cognitive deficits (affecting memory, attention, processing speed, and executive function) that improve only slightly after several months [5]. However, severe cognitive defects may affect primarily the sickest critically ill patients.

### ICU delirium and cognitive dysfunction

Guidelines for the management of pain, agitation, and delirium have highlighted the need to recognize delirium, an acute disorder that may be present in up to 80% of mechanically ventilated patients. Delirium and its duration are associated with neurocognitive dysfunction and may be associated with mortality over the first year after critical illness [5]. However, in observational studies delirium has been identified by clinicians in one-third of cases at best [6]. The ABCDEF bundle (assess, prevent, and manage pain, perform both SAT and SBT with safety screens and failure criteria, adequate **c**hoice of analgesia and sedation, delirium assessment and management, early mobility and exercise, and family engagement and empowerment) recognizes the inter-connectedness of cognitive, physical, and psychosocial issues and is attracting attention to help address these issues around recognition [7]. Data from a multi-hospital quality improvement initiative in California incorporating >6000 patients demonstrated a dose response for compliance such that for every 10% increase in compliance with the ABCDEFs, there was an independent increase in both survival and reduction in delirium/coma days even after adjusting for age, acute severity of illness, and being on or off mechanical ventilation [7].

Post-ICU psychological morbidity persists over time. One year after ICU discharge, up to one-third of patients have symptoms of depression [8], but pre-illness psychiatric history can be found in only 11–38% of patients [9]. The link between pre-illness and post-ICU psychiatric disorders is not yet completely understood.

### ICU-acquired weakness

Physical weakness, the ability to exercise and return to work, is severely impaired in more than half of ARDS survivors [3, 10]. Polyneuropathy, myopathy, and disuse atrophy can be detected early using clinical testing and electrophysiology, develop in approximately 25% of patients requiring prolonged mechanical ventilation, and are associated with increased mortality [11]. Respiratory muscle weakness, and more particularly diaphragmatic dysfunction, has been reported in about half of patients at the time of ICU admission and are associated with increased mortality [12]. Ventilation-induced diaphragmatic dysfunction occurs in half the patients after prolonged controlled mechanical ventilation or ventilation with high-level support, but can also be caused by sepsis alone (without prolonged mechanical ventilation) [13, 14]. Diaphragmatic weakness follows muscle fiber injury, atrophy, and remodeling and is associated with asynchrony, weaning failure, prolonged ventilation, and ICU and hospital readmission [15, 16]. Weakness may not be documented at clinical examination a long time after ICU discharge; however, all patients report varying levels of perceived weakness that prevents them from performing vigorous exercise [3, 10].

Studies have assessed benefits from interventions to avoid immobilization, bed rest, and muscle atrophy. Trials have evaluated early mobilization and rehabilitation, neuromuscular electrical stimulation (NMES), cycle ergometry, and functional electrical stimulation assisted cycling. In an early trial by Morris et al. [17] mobility therapy translated into reduced time to first getting out of bed and shorter ICU and hospital length of stay. The intervention was also associated with lower 1-year mortality or readmission. In a seminal trial performed at three ICUs, early physical and occupational therapy resulted in a shorter duration of mechanical ventilation and delirium and increased proportions of patients returning to pre-ICU functional status or returning home [18]. However, more recent studies have reported a lack of clinical benefits from exercise rehabilitation or intensive physical therapy [19–21]. One randomized trial of 240 patients discharged from ICU found that hospital-based rehabilitation, including increased physical and nutritional therapy plus information provision, did not improve physical recovery or quality of life, but improved patient satisfaction with many aspects of recovery [21]. However, early application of standardized mobilization has been recently reported to be effective in improving patients’ functional mobility at hospital discharge in surgical ICU patients [21].

The role of nutritional support in reducing ICU-acquired weakness remains unclear. One RCT indicated an increased weakness and delayed recovery with early parenteral nutrition [11]. Further work is needed to investigate the role of high- compared to low-dose protein combined with active and passive mobilization during the acute phase and post-acute phase of critical illness in recovery [22]. Evidence is needed to reconcile the lack of clinical benefit from nutritional interventions and the high probability that increasing substrate (protein) may actually enhance muscle proteolysis [23].

### Role of sedation

A major challenge in assessing shorter- and longer-term physical and psychological morbidity in ICU survivors is the use of early and prolonged sedation. Sedation has an effect on time to weaning, clinician’s ability to implement early mobilization, cognitive dysfunction, health-related quality of life (HRQOL), and mortality [24]. In the SLEAP trial, lack of recall of ICU events was reported by 26% of patients at day 28 [25]. However, delusional memories that have been associated with altered HRQOL and post-traumatic stress symptoms were reported in 70% at day 28. In this study, lack of recall of ICU events was independently associated with the use of midazolam and fentanyl, but delusional memories were not. Other studies reported variable results. Treggiari and colleagues conducted a randomized trial indicating that light, compared with deep sedation, reduced ICU length of stay and duration of ventilation without negatively affecting subsequent patient mental health or patient safety [26]. More studies are needed to improve our knowledge of the merits and potential harms from light sedation.

### Pain control

Specific attention to the diagnosis, prevention, treatment, and control of pain is a major component of high-quality care, as pain increases physiologic stress response, negatively affects HRQOL, and can become chronic. Development of chronic pain is associated with a number of patient-related psychological factors and predisposing factors (older age, female gender). In addition, chronic pain depends on initial pain management and is more common when pain lasts for a long time or is of high intensity or uncontrolled [27, 28]. The current US opioid crisis, however, serves as a reminder of the potential for development of opioid use disorder in the context of prolonged, prescribed analgesia. The concept of eCASH (early Comfort using Analgesia, minimal Sedatives and maximal Humane care) was recently developed [29] wherein analgesia is considered first, and sedation kept minimal, unless absolutely necessary. Better humanity of care with improved communication may minimize the need for sedation. Attention to pain control and opioid-sparing behaviors may have the potential to decrease in ICU-acquired weakness and delirium, but also to mitigate the opioid crisis and its serious moral, social, and cultural impact.

### Other long-term effects

In the frailest patients (such as those suffering acute exacerbations of COPD, heart failure, and cirrhosis), critical care illness could be a precipitating event for the late life spiral. Persistent multisystem organ dysfunction, persistent immune dysregulation, atrial fibrillation, stroke, residual end-organ damage, chronic kidney failure, exacerbation of pre-existing chronic disease, and microbiome alterations may all contribute to the increased morbidity and mortality of ICU survivors [30]. For instance, persistent elevation of IL-6 and IL-10 at ICU discharge is associated with cause-specific mortality, chiefly from cardiovascular disease (accelerated atheroma) and cancer [31], with persistent inflammation associated with muscle weakness [32]. Along the same lines, the prevalence of endocrinopathy is high after critical care illness, with potentially long-lasting (if not indefinite) alteration of cortisol, anterior pituitary hormones, or target organ hormones [33].

### Person-centered care

Critical illness triggers existential questions and invokes matters of spirituality—a core dimension of health according to the World Health Organization (http://www.medizin-ethik.ch/publik/spirituality_definition_health.htm). ICU clinicians are increasingly aware of the way in which psychological, social, cultural, spiritual, behavioral, and economic factors influence how illness affects our lives—a concept satirically labeled “personomics” [34]. Explicit incorporation of personomics and patient-centered care in educational curricula and at the bedside during direct patient care is beginning to be accorded increasing importance. Many nurses, doctors, spiritual care clinicians, and other professionals are aware of the value of presencing—being physically and emotionally available to patients and families, treating them with dignity, and helping to them address the powerlessness and the learned helplessness they often experience. Compassion has emerged among the most important attributes for critical care clinicians [35]. Acts of compassion are crucial for those likely to live as well as for the dying. Person-centered healthcare from the perspective of the critically ill means understanding “what matters most” to individuals not only while they are critically ill but also afterwards, including the relative importance of living independently, having a social role, being cognitively intact, being pain-free, and/or resuming the ability to work.

### Family-centered care

Family outcomes are also of paramount importance [36]. Increasing attention is being given to improving clinicians’ skills with regard to eliciting and exchanging information, non-verbal communication, conflict prevention and resolution, end-of-life care, and grief counseling. Unfortunately, to date, the results of interventions to improve family outcomes have been mixed.

In a single-center, before–after study design, a day-3 family conference reduced the number of days without consensus between ICU clinicians and family members and decreased ICU length of stay [37]. A multicenter, randomized trial indicated that a proactive communication strategy that includes end-of-life family conferences and a bereavement leaflet significantly reduces, in family members, symptoms of anxiety, depression, and post-traumatic stress 3 months after a patient’s death [38]. Participation of a nurse or social worker trained for 2 days to facilitate communication within the ICU team and address communication needs and conflicts has been associated with reduced symptoms of depression at 6 months, as well as reducing ICU length of stay and costs [39]. However, a multi-faceted, nurse-focused, interprofessional quality improvement intervention on end-of-life care proved ineffective in one of the largest and most generalizable studies to date [40].

In a multicenter randomized clinical trial among patients receiving 7 days of mechanical ventilation, one or two structured family meetings led by palliative care specialists (without formal palliative care consultation) demonstrated no benefits, and possibly greater post-traumatic stress symptoms in the intervention group [41]. Another trial investigated the effects of an eight-session, simulation-based communication skills intervention for internal medicine and nurse practitioner trainees on patient- and family-reported outcomes. The intervention was associated with no change in the quality of communication, but significantly increased patient depression scores [42]. Finally, increased rates of post-traumatic stress and depression symptoms were found in bereaved relatives randomized to receive a condolence letter [43]. These studies highlight the need to better identify those family members in need of intra and post-ICU interventions, to identify measures of caregiver outcomes that are responsive to intervention, and to continue to evaluate both short- and long-term outcomes of our interventions before including them in the standard of care. Finally, an extended family visitation policy in the ICU can be associated with reduced occurrence of delirium and shorter length of delirium/coma and ICU stay [44].

### Multidisciplinary approaches

For the immediate future, the research agenda is rich and challenging. Ample data on the long-term (years) adverse effects of relatively short periods (weeks) of critical illness are available. Additional trials based on the insights gained should be conducted and could be designed with reasonable equipoise. Studies to date suggest that early interventions may be beneficial, and later interventions less so. Yet, we also need to better understand whether there is a vulnerable period during which damage could result from an intervention being “too early”. Common definitions need to be better developed to create a shared glossary of terms related to ICU recovery.

Collaboration is essential between scientists and clinicians define the cerebral and neuromuscular complications of critical illness in terms of the pathophysiologic and pathobiological mechanisms. Further studies focused on early physical therapy are needed to determine the most appropriate target population who are most likely to benefit with attention paid to the specific comorbidities or diagnoses to facilitate risk stratification and to promote response to treatment. Residual axonal loss should be evaluated in patients with critical illness polyneuropathy and persistent physical impairment and fatigue. Altered muscle regeneration should be examined in patients with critical illness myopathy and persistent physical impairment and fatigue. These collaborative efforts may lead to promising novel treatments such as mesenchymal cell therapy [45].

Perhaps most importantly, effective interventions must be developed, tested, and implemented, at various levels, to improve patients’ and caregivers’ abilities to cope with critical illness and its sequelae (prevent tissue impairment, rehabilitate activity limitations, compensate limitations, or adapt with reduction in quality of life). Some evidence exists that rehabilitation should start early during critical illness, but modalities of rehabilitation remain to be tested and clarified. Although additional studies are needed to reconcile uncertainties about early physical and occupational therapy, addressing the detrimental effects of immobilization and sedation is urgent as they are already known to impair physical and cognitive status. Whether weaning strategies should be tailored based on respiratory muscle monitoring prior to extubation requires further evaluation. More research is needed to further document the (non)recovery of the neuro-endocrine alterations that occur during critical illness and on how these correlate with/affect the legacy of an ICU stay.

### Future perspectives and research agenda

In the near future, we are committed to sharing practical strategies to increase the rigor, efficiency, and impact of individual observational studies and randomized trials. We are also committed to ensuring that key training and relevant practical experiences for junior investigators, clinical researchers, and scientists from diverse disciplines are encouraged to help build more and better multicenter and multinational collaboration. In future studies, proper use of statistical methods will be required (i.e., informative censoring, the competing risk and time-dependent nature of covariates, such as the occurrence of infection or delirium). Moreover, publicly disclosing adjustment models with the coefficients and the variables used will help reconcile apparent paradoxes in the literature. Lastly, access to data from large prospective studies for secondary analysis, single patient metaanalysis, etc. may also provide additional evidence to guide practices at the bedside. Long-term chronic pain, addiction, and dependencies need to be studied in ICU survivors using valid tools that help us understand predictors of ICU-related opioid dependency, and the complex relationship between post-ICU chronic pain and opioid use and dependency. Moreover, multimodal analgesic interventions to avoid ICU opioid dependency and empirically validated psycho-social protocols to address opioid use disorders are much needed. Also, because delirium has a longer-term impact on mortality and cognitive function, and because drug therapy alone will likely be insufficient to prevent or treat it fully, priority should be given to testing on a large scale the clinical impact of the ABCDEF bundle and the ICU Liberation Initiative, as well as how the overall management of the human body affects that of the brain [6].

## Conclusions

Systematic post-ICU follow-up may assist patients and family caregivers with their perspective of recovery. They need to know what to expect, so as to understand the path to recovery, to acknowledge uncertainties, and to be equipped to adjust to the later sequelae. More research is needed to help determine what interventions can usefully be implemented in ICU follow-up clinics and the appropriate physical intervention programs. Furthermore, patients’ and family members’ experiences should be used to guide effective interventions to provide the best comfort and holistic care, to better address the needs of patients and their families, and improve our ability to alleviate the post-ICU burden for surviving patients and their loves ones, as well as the grief symptoms of bereaved family members. Finally, elements of palliative care need to be integrated early into the care of the sickest critically ill patients, whatever the goals of care, with the sole aim of improving the ICU experience for those who are dying and for those who will face the challenging period of post-ICU recovery. ICU clinicians should be trained in structured communication approaches, multidisciplinary interventions to increase the emphasis on patient values for supporting preference-sensitive decisions in the face of uncertainty, delivery of support aids such as printed materials and diaries, and dignity-conserving care.

## Abbreviations

ARDS: Acute respiratory distress syndrome
ICU: Intensive care unit
